# Development of the Canadian Coalition for Seniors' Mental Health Canadian Guidelines on the Assessment and Management of Behavioural and Psychological Symptoms of Dementia

**DOI:** 10.1002/alz.089615

**Published:** 2025-01-09

**Authors:** Stacey E Hatch, Jennifer Watt, Marie‐Andrée Bruneau, Vivian Ewa, Sid Feldman, Yael Goldberg, Zahra Goodarzi, Nathan Herrmann, Debbie Hewitt Colborne, Alexandre Henri‐Bhargava, Zahinoor Ismail, Julia G Kirkham, Sanjeev Kumar, Krista L. Lanctôt, Wade Thompson, Jennifer Porter, Dallas Seitz

**Affiliations:** ^1^ Hotchkiss Brain Institute, University of Calgary, Calgary, ON Canada; ^2^ CIHR Institute of Aging, Ottawa, ON Canada; ^3^ Unity Health Toronto, Toronto, ON Canada; ^4^ University of Montreal, Montreal, QC Canada; ^5^ University of Calgary, Calgary, AB Canada; ^6^ The College of Family Physicians of Canada, Mississauga, ON Canada; ^7^ Baycrest Health Sciences, Toronto, ON Canada; ^8^ Temerty Faculty of Medicine, University of Toronto, Toronto, ON Canada; ^9^ Hotchkiss Brain Institute, University of Calgary, Calgary, AB Canada; ^10^ Sunnybrook Health Sciences Centre, Toronto, ON Canada; ^11^ Behavioural Supports Ontario Provincial Coordinating Office, North Bay, ON Canada; ^12^ Neil and Susan Manning Cognitive Health Initiative, University of British Columbia, Victoria, BC Canada; ^13^ Centre for Addiction and Mental Health, Toronto, ON Canada; ^14^ University of Toronto, Toronto, ON Canada; ^15^ University of British Columbia, Vancouver, BC Canada; ^16^ University of Calgary, Calgary, ON Canada

## Abstract

**Background:**

Behavioural and psychological symptoms in dementia (BPSD) are common, can be distressing for persons living with dementia (PLWD), and challenging for caregivers and clinicians. There are few updated clinical practice guidelines available to assist clinicians with the assessment and management of BPSD, including deprescribing of medications. The Canadian Coalition for Seniors’ Mental Health (CCSMH) developed Canadian clinical practice guidelines on the assessment and management of BPSD to address these needs.

**Methods:**

We used the Guideline International Network process for development of the BPSD guidelines. A multidisciplinary guideline panel was formed including representatives from geriatric psychiatry, geriatric medicine, family medicine, nursing, pharmacy, and psychology. Surveys identified priority topic areas for the guidelines. A review of existing guidelines, systematic reviews and primary evidence reviews was undertaken to identify evidence to create the guideline recommendations. The guideline panel members framed guideline questions using the PICO format (patient, intervention, comparator, and outcome). Guideline recommendations were created using the Grades of Recommendation, Assessment, Development, and Evaluation (GRADE) tool and assigned a strength of recommendation (strong or conditional) and quality of evidence (high, moderate, low, and very low). The final guideline recommendations were voted on by the guideline panel with consensus considered to be 80% agreement.

**Results:**

The guideline panel met a total of 14 times. A total of 74 recommendations reached consensus and were included in the guideline. These included recommendations on general principles of BPSD assessment and management, BPSD diagnosis, detection, and pharmacological and non‐pharmacological management of BPSD syndromes. The following topic areas and recommendations per topic were: general principles of BPSD assessment and management (n = 11), agitation (n = 30), psychosis (n = 5), depression and depressive symptoms (n = 13), anxiety (n = 6), sexual expressions with associated risk (n = 4), and deprescribing (n = 5).

**Conclusions:**

The CCSMH Canadian Guidelines on the Assessment and Management of BPSD provide healthcare providers with recommendations based on current evidence from a Canadian context. These guidelines will help inform care for the growing number of people living with dementia who may be affected by BPSD.

